# Studying the Efficiency of Waiting Time in Outpatient Pharmacy

**DOI:** 10.1016/j.mex.2020.100913

**Published:** 2020-05-13

**Authors:** Arwa Alodan, Ghada Alalshaikh, Hadeel Alqasabi, Sara Alomran, Abdelhakim Abdelhadi, Bandar Alkhayyal

**Affiliations:** Prince Sultan University

**Keywords:** Outpatient Pharmacy, Quality Assurance, Waiting time

## Abstract

In general, the pharmacy is the last department to be visited for outpatient in the hospitals, and therefore its efficiency is directly linked to patients’ satisfactions and more important the reputation of the entire hospital. The study here is based on Medical City that is located in Riyadh, Saudi Arabia. It serves patients from all over Saudi Arabia. The aim of the study is to improve the efficiency of waiting time of outpatient pharmacy based on the problems that have been observed using management quality tools and techniques. After analyzing the data for the current situation, then trying to propose changes for improvement in system efficiency. Results showed that by proposing automated waiting system with automated prescriptions, patient categorization, reduce the unclaimed prescriptions, and modify the pharmacy's layout. All of that will help in reducing the waiting time as well as increasing the patients' satisfaction which will lead to improve the pharmacy's efficiency. From reviewing the literature, it concludes that applying management quality tools and techniques will tremendously improve the quality of services in healthcare systems. The statistical analysis presented shows some outliers points when serving patients which were studied and recommendations were proposed. This is new approach to enhance the quality of healthcare management and leads to increase in the efficiency of the outpatient pharmacies.

## Graphical abstract

Customers satisfaction in the outpatient pharmacy using management quality tools (before and after).

**Figure:**
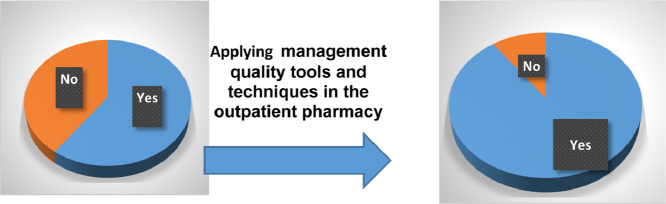

In the case of outpatients’ pharmacy, the improvement in the outpatient pharmacy efficiency includes:

• Reducing patients’ waiting time
• Increase customers’ satisfaction
• Improving the workflow

Specifications table

**Table:** 

Subject Area:	Engineering
More specific subject area:	*Outpatient pharmacy customers’ satisfaction*
Method name:	Application of management quality tools and techniques in healthcare
Name and reference of original method:	*If applicable, include full bibliographic details of the main reference(s) describing the original method from which the new method was derived.*
Resource availability:	*N/A)*

## Introduction

The Medical City under study is located in Riyadh, Saudi Arabia and serves patients from all over Saudi Arabia since it is one of the advanced centers in the Middle East. The research focuses on Medical City Outpatient Main Pharmacy, which aims to improve the quality of services provided to patients and enhance their experience. After conducting a survey, the following information has been discovered. First, the pharmacy serves all outpatient clinics and the average waiting time for the patients is between *90 to 120* minutes. Second, prescriptions are written manually by doctors which might cause some difficulties to the pharmacists. The number of medicines prescribed is between *1500 to 1800* per day. Third, the size and layout of the pharmacy does not contribute to the number of prescriptions being prepared and the number of patients. Finally, a whole pharmacy storage is dedicated to the unclaimed prescriptions. Our goal is to decrease the waiting time, decrease the number of unclaimed prescriptions, and categorize the patients as following: long prescriptions patients, short prescriptions patients, special needs & low immunity patients and refill patients. In short, the paper aims to have more accuracy in prescriptions and communications between doctors and pharmacists which will improve the efficiency.

## Problem Description

The pharmacy's major problems are as following:

1- Prescriptions are written manually by doctors which might be difficult to read by pharmacists. Also, the medicine might not be available at the pharmacy without the doctor's notice.
2- Lack of patient categorization, all types of patients are served from the same window. Each type of patient consumes the pharmacist's time differently. For example, the special needs & low immunity patients require more time since the pharmacists needs to explain thoroughly the prescription. On the other hand, the refill patient service should be by itself and fast.
3- The pharmacy serves all outpatients from all clinics which results long patient's waiting time at the pharmacy.
4- High number of unclaimed prescriptions which requires storage and consumes the pharmacist's time.
5- The pharmacy's space is small and tight compared to the number of patients served and number of medicine stored which is uncomfortable for both patients and pharmacists.

## Literature Review

Improving healthcare quality has been a great concern for academics, professionals, and practitioners of healthcare services. Many studies in the literature investigate healthcare service quality and related issues such as patients’ dissatisfaction due to the long waiting time. In this study, the literature reviews are related to define and analyze the problems at hand.

According to a joint study by the World Health Organization (WHO) and the World Bank in 2018, poor quality health services are holding back progress on improving health in countries at all income levels. WHO also defined the quality of healthcare as:

*“The extent to which healthcare services provided to individuals and patient populations improve desired health outcomes. In order to achieve this, health care must be safe, effective, timely, efficient, equitable and people-centered.”* P30. [7]. The meaning of timely in the definition is reducing delays in providing and receiving health care, as described in the study.

A study by Mohammad Mosadaghard [4], explores health stakeholders’ perspectives on service quality in an attempt to establish a comprehensive definition of quality that can meet all stakeholder expectations in the health care system. Identifying quality attributes can help all parties establish and maintain continuous quality improvement programs. Stakeholders in this study include clients, professionals, managers, policymakers, and payers. After an extensive review of the literature, many definitions of healthcare quality pertaining to each of the stakeholders were found.

According to the study, most of the definitions in the study can be placed into two groups:

1. Healthcare services that meet predetermined specifications and standards. In this case, quality can be defined as “conformance to specifications requirements or standards”
2. Healthcare services that meet customer expectations in which quality is defined as “satisfying customers’ expectations and needs”.

The first group focuses on the provider's side and internal factors to be considered such as accuracy, reliability, and efficacy to define and improve quality. The second group focuses on the customers’ side and external factors such as effectiveness, empathy, safety, and affordability, all of which are considered as important quality factors. The scholar uses pluralistic evaluation methods to represent all stakeholders’ views. The study found that healthcare quality has a different meaning for each group. Therefore, managers, practitioners, and policymakers must take the quality dimensions of each group into consideration when evaluating and improving the quality of health care services to meet all stakeholders’ needs and expectations.

Another study by Abdelhadi & Shakoor [1] was conducted in Abha city, southwest of Saudi Arabia, to measure the service quality provided by public health hospitals. The research took place in the inpatients and outpatient pharmacies at a large public regional hospital. The study implemented Lean Manufacturing technique to evaluate and improve service quality and reduce the waiting time in both pharmacies. Lean Manufacturing or Toyota's Production System (TPS) is a management philosophy utilized by Toyota Motors Corporation in Japan. TPS technique focuses on eliminating waste, solving problems, enhancing workers' partnership, and continuing process improvement. The technique is also used to improve quality in many other services and industries such as healthcare. The TPS approach is based on principles that focus on understanding the wastes or non-value-added steps in the system, eliminating them, and responding to customers’ needs to arrive at perfection. TPS is devised as an improvement tool that enhances efficiency and quality of service to reduce the time needed for service delivery by comparing the efficiency between the two pharmacies. The data was collected by observing the workflow in the two pharmacies for a week.

In their paper, the researchers used a metric tool in lean manufacturing called Takt time to measure the efficiency of both pharmacies. The results showed that the inpatient pharmacy was more efficient than the outpatient pharmacy, as the time of filling a prescription is average and close to the ideal situation. The paper found that the adoption of lean manufacturing principles could be used as an efficiency measure for healthcare services quality by comparing the efficiency between two or more departments within the system. The findings of this paper are of great help for pharmacy managers to identify the causes of variations in efficiency between departments and make corrections to solve the problems.

Many studies in the literature adopted different managerial tools and techniques to solve healthcare services problems and improve quality. For instance, a study conducted by Arafeh et al. [8] implemented Six Sigma processes as an improvement methodology to reduce patients’ waiting time in an outpatient pharmacy located in a local hospital specialized in cancer treatment in Pakistan. The study found various improvement opportunities that can reduce patients waiting time by 50%.

Suss et al. [6] undertook a patient flow project to reduce the waiting time and improve efficiency spends in pharmacy queues. They proposed solutions to the problems and provided a framework to evaluate pharmacy performance based on simulations.

Ahmad et al. [2] studied the patient waiting time and doctor consultation time in a primary healthcare clinic and to formulate strategies for improvement. The data were collected and entered using statistical software SPSS for analysis. They have identified that in order to improve the waiting and consultation time they should increase the number of staff at the registration counter, enforcing the staggered appointment system for follow-up patients and improving the queuing system for walk-in patients.

Rim et al. [5] implemented a quality assessment to an outpatient pharmacy services call center by the following: (1) decreasing the call rejection rate, (2) improving the speed of answer, (3) increasing first-call resolution, (4) centralizing all specialty pharmacy and prior authorization calls, (5) increasing labor efficiency and pharmacy capacities, (6) implementing a quality evaluation program, and (7) improving workplace satisfaction and retention of outpatient pharmacy staff. The design and implementation have significantly improved the health system's patient experiences, efficiency, and quality.

## Input Data and Statistical Analysis

Data analysis, which is the process of inspecting, presenting and reporting data in a way that is useful for readers. Observation of outpatient main pharmacy waiting time data took place on Tuesday 19th of November 2019 between 8:00 – 9:00 a.m. by one of our members in terms of waiting time per prescription. Tuesday was selected on purpose to represent the worst case scenario because it is the peak day of the weak according to the pharmacy's director, the time was observed from the opening of the pharmacy to the first hour of their peak time. Table 1. shows the time for the first 50 tickets that were issued during that time.

**Table 1:** Data collected in a peak day from outpatient pharmacy

Ticket #	Waiting Time (hr.)	Processing Time(hr.)	Cycle Time(hr.)
1	0.33	0.12	0.45
2	0.34	0.13	0.47
3	0.37	0.15	0.52
4	0.38	0.15	0.53
5	0.42	0.13	0.55
6	0.48	0.13	1.01
7	0.5	0.14	1.04
8	0.52	0.16	1.08
9	0.55	0.16	1.11
10	0.58	0.16	1.14
11	1	0.17	1.17
12	1.01	0.17	1.18
13	1.02	0.19	1.21
14	1.06	0.2	1.26
15	1.08	0.21	1.29
16	1.08	0.22	1.3
17	1.1	0.21	1.31
18	1.11	0.21	1.32
19	1.12	0.21	1.33
20	1.12	0.22	1.34
21	1.13	0.22	1.35
22	1.14	0.22	1.36
23	1.16	0.21	1.37
24	1.16	0.23	1.39
25	1.2	0.2	1.4
26	1.21	0.19	1.4
27	1.23	0.2	1.43
28	1.22	0.22	1.44
29	1.22	0.22	1.44
30	1.22	0.22	1.44
31	1.22	0.23	1.45
32	1.22	0.23	1.45
33	1.23	0.25	1.48
34	1.25	0.37	2.02
35	1.25	0.27	1.52
36	1.26	0.27	1.53
37	1.23	0.3	1.53
38	1.24	0.32	1.56
39	1.26	0.31	1.57
40	1.26	0.31	1.57
41	1.3	0.31	2.01
42	1.32	0.42	2.14
43	1.32	0.33	2.05
44	1.32	0.33	2.05
45	1.37	0.31	2.08
46	1.43	0.32	2.15
47	1.44	0.34	2.18
48	1.47	0.35	2.22
49	1.49	0.34	2.23
50	1.48	0.37	2.25

As we can notice from the above observations that the long waiting time problem occurs from the first ticket and it has been increasing linearly until the end of the first period. This due to conjunction that happens when patients start coming. Therefore, a serious improvement needs to happen for their waiting time.

**Figure 1:**
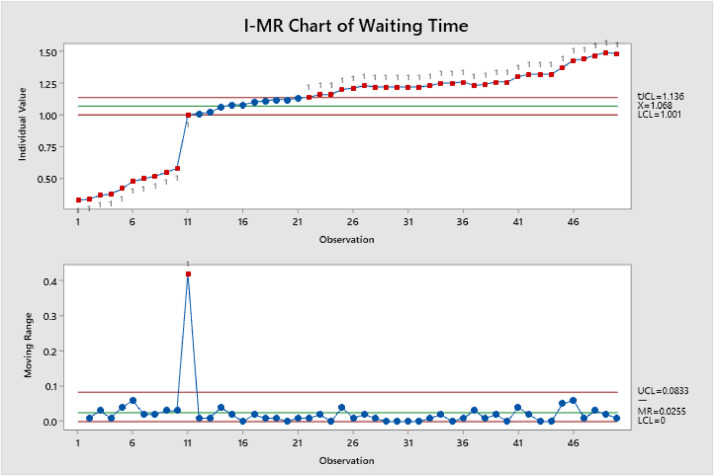
Shows I-MR of Waiting Time

**Figure 2:**
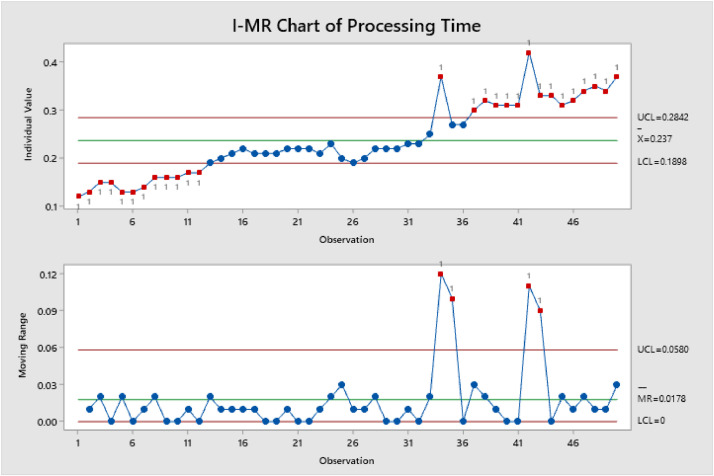
Shows I-MR of Processing Time

**Figure 3:**
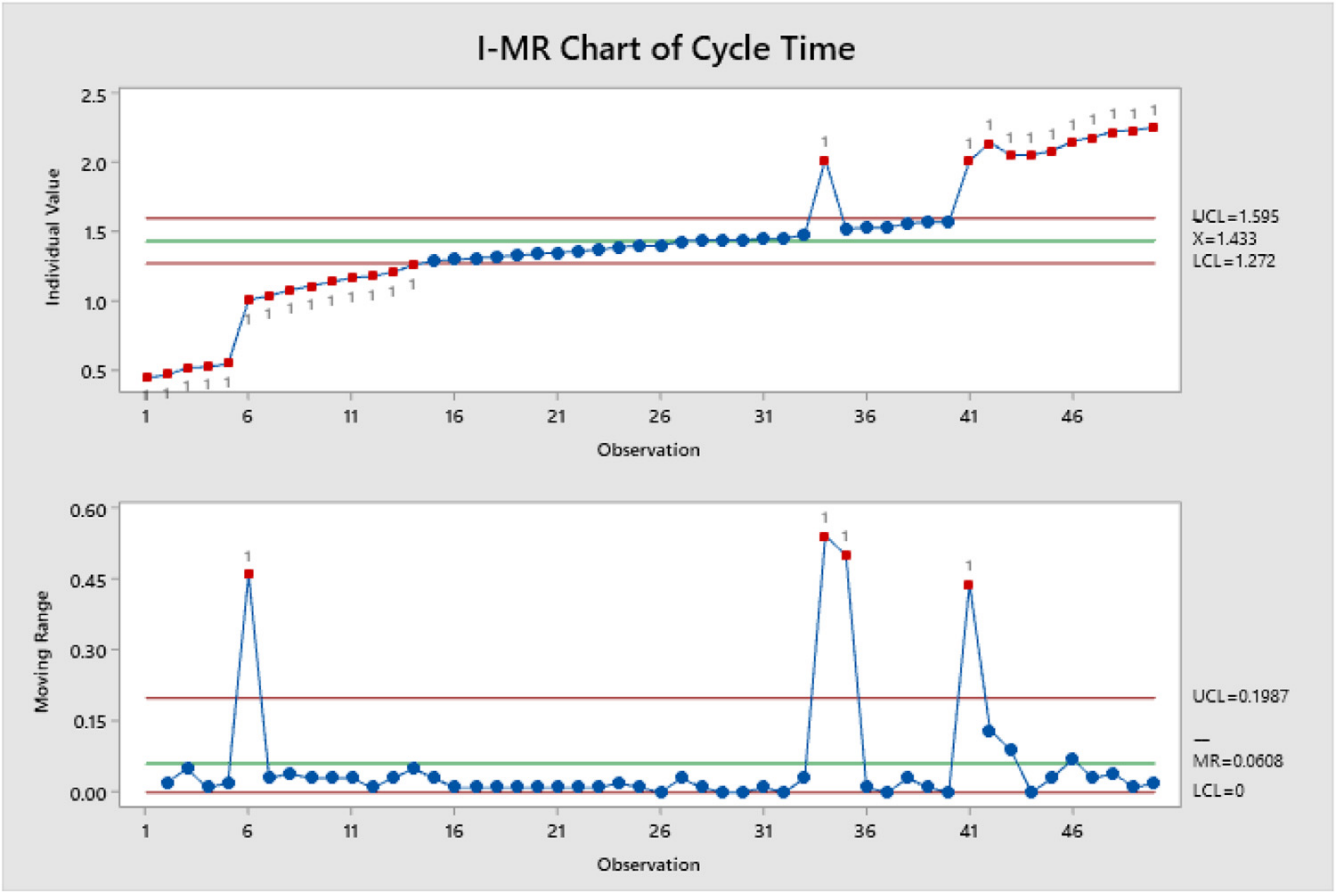
Shows I-MR of Cycle Time

Statistical analysis is used in order to gain an understanding of a larger population by analyzing the information of a sample. Therefore, Figure 1, Figure 2, Figure 3 show the control charts of Waiting Time, Processing Time, and Cycle Time. Where is waiting time, is the time that the patients waited to be called, and processing time where patients prescriptions were under preparation. Cycle time is the time patients spend on the pharmacy, from entering to exit.

Minitab 19 was used for statistical analysis, and it shows that there is linear increasing from the beginning of the cycle. For example, #34 was out-of-control and after the inspection the patient had more prescriptions than others, also some patients they need more doctors’ approval so it takes more than usual.

On Figure 1, Figure 2, Figure 3, an individuals and moving range (I-MR) chart were used to determine if a process is stable and predictable, this creates a picture of how the system changes over time. The individual (I) chart displays individual measurements. The moving range (MR) chart shows variability between one data point and the next. Individuals and moving range charts are also used to monitor the effects of process improvement theories.

## Results and Discussion

Results showed that by proposing automated waiting system with automated prescriptions, where doctors can send prescriptions over an online system; this will enhance communication between doctor and pharmacist, save time and effort, as well as will increase the efficiency of the service [3]. Also, having patient categorization, where not all types of patients are served from the same window. By categorizing each type of patient in a need base, for example, special needs & low immunity patients require more time since the pharmacists needs to explain thoroughly the prescription, so they will have special privacy window, and the refill patient service should be by itself since its fast and they can request the refill over phone and/or online. This as well will reduce the unclaimed prescriptions due to the short waiting time, and since most of the unclaimed prescriptions were due to the long waiting time. Finally, it has been discussed with the Outpatient Main Pharmacy the idea of modifying the pharmacy's layout. Where and since they have 7 floors, that each mini pharmacy will serve each floor with load medication that common on that floor, since each floor has its own department. This will reduce the time, and will increase the efficiency since they will be a better communication between the pharmacy and the doctors. In this case, the main one on the ground floor will focus on refill services and special needs. This proposal can reduce the waiting time to a maximum of 15-20 minutes per prescription. The new layout plan was discussed and they can room on each floor to develop that plan. In short, all of that will help in reducing the waiting time as well as will increase the patients' satisfaction, which is our main goal on quality. After analyzing the data for the current situation for the Outpatient Main Pharmacy, it concludes that it needs changes for improvement in system efficiency.

## References

[1] Abdelhadi A., Shakoor M. (2014). Studying the efficiency of inpatient and outpatient pharmacies using lean manufacturing. Leadership in Health Services.

[2] Ahmad B.A., Khairatul K., Farnaza A. (2017). An assessment of patient waiting and consultation time in a primary healthcare clinic. Malaysian family physician: the official journal of the Academy of Family Physicians of Malaysia.

[3] Kuperman G.J., Bobb A., Payne T.H., Avery A.J., Gandhi T.K., Burns G., Classen D.C., …, Bates D.W. (2007). Medication-related clinical decision support in computerized provider order entry systems: a review. Journal of the American Medical Informatics Association: JAMIA.

[4] Mohammad Mosadeghrad A. (2013). Healthcare service quality: towards a broad definition. International journal of health care quality assurance.

[5] Rim M.H., Thomas K.C., Chandramouli J., Barrus S.A., Nickman N.A. (2018). Implementation and quality assessment of a pharmacy services call center for outpatient pharmacies and specialty pharmacy services in an academic health system. The Bulletin of the American Society of Hospital Pharmacists.

[6] Suss S., Bhuiyan N., Demirli K., Batist G. (2017). Toward implementing patient flow in a cancer treatment center to reduce patient waiting time and improve efficiency. Journal of oncology practice.

[7] World Health Organization (2018). OECD & International Bank for Reconstruction and Development/The World Bank. Delivering quality health services: a global imperative for universal health coverage.

[8] Arafeh M., Barghash M., Sallam E., AlSamhouri A. (2014). Six Sigma applied to reduce patients’ waiting time in a cancer pharmacy. Int. J. Six Sigma and Competitive Advantage.

